# Alanine Aminotransferase Elevation at Diagnosis of Youth‐Onset Type 2 Diabetes: Prevalence, Predictors, and One‐Year Outcomes

**DOI:** 10.1002/edm2.70211

**Published:** 2026-04-11

**Authors:** Sean DeLacey, Wenya Chen, Adesh Ranganna, Siyuan Feng, Mark Fishbein, Monica Bianco

**Affiliations:** ^1^ Department of Pediatrics Ann & Robert H. Lurie Children's Hospital of Chicago Chicago Illinois USA; ^2^ Department of Pediatrics Northwestern University Feinberg School of Medicine Chicago Illinois USA

**Keywords:** liver, metabolic associated fatty liver dysfunction, type 2 diabetes

## Abstract

**Introduction:**

Metabolic dysfunction‐associated steatotic liver disease (MASLD) and Type 2 Diabetes (T2D) are components of insulin resistance, but the prevalence of MASLD at the diagnosis of youth‐onset T2D is unknown. We aimed to describe the prevalence of alanine aminotransferase (ALT) elevation, a biomarker for MASLD, in youth‐onset T2D at diagnosis and after one year and investigate factors associated with ALT elevation.

**Methods:**

A single‐centre retrospective cohort study was conducted of patients (age ≤ 21 years) diagnosed with T2D between 1/1/2010 and 31/12/2021, with ALT available at diagnosis. ALT elevation was defined as being greater than 1.5 times the upper range of normal based on patient sex.

**Results:**

In the cohort (*n* = 438), 58% of patients had ALT elevation at T2D diagnosis. Hispanic patients had higher odds of ALT elevation than non‐Hispanic Black patients (*p* < 0.001), and lower HbA1c at diagnosis was associated with higher odds of ALT elevation (*p* < 0.001). Among patients followed for one year (*n* = 141), the prevalence of ALT elevation decreased from 65% to 47%. ALT at diagnosis was not associated with a change in Haemoglobin A1C (HbA1c) from diagnosis to one year of follow‐up, nor was HbA1c at diagnosis associated with a change in ALT.

**Conclusions:**

ALT elevation is common at T2D diagnosis in youth. HbA1c is negatively associated with ALT elevation at diagnosis, but ALT and HbA1c at diagnosis did not impact the change in the corresponding marker at one year. The prevalence of elevated ALT varies between groups, and analysis of disease course within subpopulations is pertinent.

## Graphical abstract

A single‐centre retrospective review of youth‐onset Type 2 diabetes at diagnosis and one‐year follow‐up, showing a high prevalence of alanine aminotransferase (ALT) elevation at both time points. ALT elevation at diagnosis was associated with Hispanic ethnicity and lower HbA1C, but there was no clinically significant association between ALT elevation at diagnosis and HbA1C trend over time.

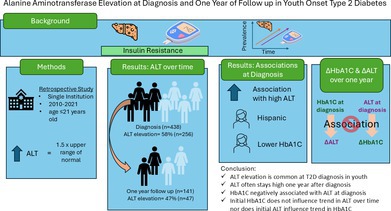

## 1 Introduction

Rising obesity and its associated insulin resistance represent a major health crisis for youth in the United States. Each year in the United States, approximately 5000 new children are diagnosed with Type 2 diabetes (T2D), and nearly 1 in 5 adolescents aged 12–18 years have prediabetes [1, 2]. Youth‐onset T2D has risen over time as the prevalence of childhood obesity exponentially grows [1]. Youth‐onset T2D is a distinct entity from adult disease with an elevated risk of subsequent complications, treatment failure, and beta‐cell decline [3, 4, 5].

Like T2D, the prevalence of metabolic dysfunction‐associated steatotic liver disease (MASLD) is increasing, with a prevalence of 18.5% in youth 12–21 years old in the United States [6]. Metabolic dysfunction‐associated steatohepatitis (MASH) is the second most common (and the most quickly rising) indication for liver transplant in the adult population and is a pressing problem for both adults and children [7]. While knowledge of the natural history of MASLD in children is limited, it is estimated that one‐third of those with MASLD will experience disease progression to MASH and worsening fibrosis. T2D has been reported to be the strongest risk factor for fibrosis progression in children [8, 9].

Indeed, evidence suggests that MASLD and insulin resistance are interlinked. In paediatric MASLD, the estimated prevalence of prediabetes and T2D is 23.4% and 6.5%, respectively [10]. In paediatric T2D, the prevalence of elevated alanine aminotransferase (ALT) described in one study was 48% [11]. The pathophysiology of both diseases is linked: chronic overnutrition can lead to increased oxidative stress and reactive oxygen species that create insulin resistance in target tissues like skeletal muscle, adipose tissue, and the liver [12]. Insulin resistance in both adipocytes and hepatocytes leads to unsuppressed lipolysis and an increase in de novo lipogenesis. The subsequent rise in toxic metabolites (glucotoxicity and lipotoxicity) causes increased oxidative stress and inflammation, leading to liver injury [13, 14, 15]. Similarly, as liver inflammation occurs, it can lead to increased hepatic insulin resistance, and hence a failure for insulin to suppress hepatic gluconeogenesis and exacerbate glucotoxicity that impairs beta cell function [16, 17]. However, the interrelationship between these two diseases is incompletely understood in the paediatric and young adult population.

Current T2D research in adults has explored risk factors and comorbidities that contribute to the heterogeneity of the disease, and has discovered that severe‐insulin resistant diabetes (SIRED) results in the highest risk for diabetic kidney disease and MASLD [18]. While MASLD is common in paediatric T2D, its impact on the progression of glycemic control over time remains unclear. In addition, the clinical features that predispose children to MASLD at the time of T2D diagnosis have not yet been identified in youth. Understanding how MASLD and T2D evolve together and what patient or care characteristics, such as medications used or residual beta cell function, influence their progression is an important first step in improving the care of youth with T2D.

The presence of MASLD is typically established in children using surrogate markers such as serum ALT and non‐invasive imaging techniques such as ultrasound and MRI due to the invasiveness and risks of liver biopsy [19]. As such, we aim to characterise the patterns of ALT elevation as a marker of MASLD at diagnosis of T2D in youth and over the first year of treatment, as well as explore demographic features associated with both the elevation at diagnosis and the change in ALT level over time. We hypothesised that ALT elevation at diagnosis would be associated with an upward trend in HbA1c one year after diagnosis. This study characterises the relationship between T2D and MASLD and may potentially help determine the need for hepatology referral in youth with T2D and elevated biomarkers (ALT) on screening to monitor for progression to MASH.

## 2 Materials and Methods

### 2.1 Study Population

We conducted a retrospective cohort study of patients seen within the Ann & Robert H. Lurie Children's Hospital of Chicago system diagnosed with T2D between 1/1/2010 and 31/12/2021. We built upon data from a prior retrospective study evaluating the prevalence of paediatric T2D over time in relation to the COVID pandemic done within the same institution, and hence many of the methods for data collection were the same but modified for the current aims [20]. T2D was defined by a glycated haemoglobin (HbA1c) greater than or equal to 6.5% (48 mmol/mol), OR oral glucose tolerance test results (fasting glucose > 126 mg/dL OR 2‐hour glucose > 200 mg/dL), OR a random blood glucose of > 200 mg/dL with consistent symptomatology (Table S1). Clinical data were extracted from electronic medical records (EMR). This study was conducted in accordance with the Declaration of Helsinki and was approved by the Lurie Children's Hospital Institutional Review Board prior to any data collection (IRB 2022–5342).

All patients seen at the institution or any of its satellite locations with a clinical diagnosis of T2D were eligible for inclusion if data from their initial diagnosis encounter were available for review and occurred within the study period. Records were screened by using the following criteria: International Classification of Diseases (ICD) codes for diabetes (ICD‐9 code 250.00, or ICD‐10 codes E13.9 or E11.9), an encounter 1/1/2010–31/12/2021, and age ≤ 21 years old at diagnosis. The initial screen yielded 547 separate patients. The charts were screened and validated by two members of the research team (S.D. and M.B.) to confirm the clinical diagnosis of T2D and ensure they did not meet any exclusion criteria. Exclusion criteria included: No liver chemistries within two months of diagnosis of T2D, diagnosis of other forms of diabetes mellitus (such as type 1 diabetes, medication associated diabetes, cystic fibrosis‐related diabetes, or maturity onset diabetes of the young (MODY)), > 1 diabetes autoantibody positive, complex medical history potentially contributing to diabetes, a BMI < 85% for age and sex using the Centers for Disease Control and Prevention data, other forms of liver disease, or other medications thought to be contributing to liver disease. Those with a BMI < 85% were excluded to decrease the chance that children with other types of diabetes, such as antibody‐negative Type 1 diabetes or MODY, were included in the analysis. After screening for inclusion and exclusion criteria, 438 patients were included for analysis. Follow‐up analyses only included those that had liver chemistries done between 9–15 months of diabetes diagnosis (*n* = 141).

### 2.2 Methods

All laboratory data were included if they could be accessed through internal or external medical records. If a lab value was reported as greater than or less than a certain value, that value was used in the data entry. If more than one set of liver labs was available within two months of diagnosis, those closest to the date of diabetes diagnosis were used. Similarly, follow‐up ALT and HbA1c levels were selected based on how close they were to 12 months after the initial diabetes diagnosis date.

We chose to focus on serum ALT, as it is endorsed by the North American Society of Paediatric Gastroenterology, Hepatology and Nutrition (NASPGHAN) as the best screening test for fatty liver disease in children [21]. Additionally, ALT has been used as a surrogate for MASLD in other population studies [22]. We defined ALT elevation as greater than 1.5 times the upper limit of normal based on sex in population‐wide studies; specifically, the upper limit of normal is defined as 22.1 IU/L for females and 25.8 IU/L for males, based on prior NHANES studies (Table S1) [23]. Further supporting the validity of our chosen threshold, previous research demonstrated that an ALT cutoff of > 40 IU/L has a sensitivity of 44% and a specificity of 89% in detecting MASLD in an obese paediatric population [24]. We did not analyse results by age because the age group was concentrated in the 10–15‐year‐old range, and we were unable to adjust for pubertal status. A change in ALT value over time has also been used as a marker for liver disease in prior studies [25]. AST/ALT ratio has also been used as a proxy for liver disease in those with MASLD in the past, and was included as well, and trended over time as another non‐invasive proxy used for assessment of liver disease. A cutoff of 1 for the AST/ALT ratio was chosen because it is suggested as a cutoff for more advanced disease screening by NASPGHAN [26]. However, we chose to focus on ALT elevation as the primary marker of MASLD because there is more precedent for this in paediatric literature.

Imaging study reports (ultrasound and MRI) were reported when available and performed within the study interval. Any mention of “fat” or “steatosis” on liver imaging was noted categorically by study staff based on the report (present or absent). The percentage of patients referred to a hepatologist for further evaluation was also recorded.

BMI percentiles were calculated according to the Centers for Disease Control and Prevention standard growth charts [27, 28]. Sex was defined by sex assigned at birth as recorded in the EMR. Information on gender was not collected. Racial and ethnic group information and insurance information were collected from the EMR based on the predefined categories within the EMR.

### 2.3 Statistical Analysis

Patient demographics and clinical characteristics were summarised using frequencies (*n*) and percentages (%) for categorical variables, means with standard deviations (SD) for normally distributed continuous variables, and medians with interquartile ranges (IQR) for non‐normally distributed continuous variables. The Shapiro‐Wilks test was used to check the normality of continuous data. To explore the demographic and clinical factors associated with ALT elevation at diagnosis, we estimated unadjusted odds ratios (OR) and 95% confidence intervals (CI) using logistic regression. For patients who had liver labs available within 9–15 months of diagnosis, we examined differences in BMI, ALT, and HbA1c at diagnosis and one year of follow‐up, using the paired *t*‐test or Wilcoxon signed‐rank test for continuous variables, and McNemar's test for categorical variables.

Multiple linear regression models were used to evaluate: (1) the association between ALT at diagnosis and BMI z‐score at diagnosis on one‐year change in HbA1c; (2) the association between ALT at diagnosis and HbA1c at diagnosis on one‐year change in BMI z‐score; and (3) the association between BMI z‐score at diagnosis and HbA1c at diagnosis on one‐year change in ALT. All models were adjusted for age, sex, race/ethnicity, and metformin use. Covariates were selected because of the known association between each variable with both HbA1C, BMI, and ALT from previous literature [18, 29, 30, 31, 32, 33, 34, 35]. Model assumptions were assessed using residual diagnostics. P‐values < 0.05 (two‐sided) were considered statistically significant. Statistical analyses were conducted in R version 4.1.0 within RStudio version 1.2.1335.

## 3 Results

A total of 438 patients were included in the analyses of patients at diagnosis, and 141 of these patients were included in follow‐up analyses with a median follow‐up duration of 1.04 years (Table 1, Table 3). Patients' ages ranged from 9–19 years old, with a median age of 14.19 years; 2% of patients were < 10 years old, 29% 10–12 years old, 48% 13–15 years old, 19% 16–18 years old, and 2% > 18 years old. The sample was equally female and male (49.8% female). At diagnosis, the mean BMI z‐score was 2.42, and the mean BMI was 35.58 kg/m2. Additionally, the median HbA1c was 8.3% at diagnosis (IQR 7.00–10.70).

**TABLE 1:** Descriptive statistics at diagnosis for all patients.

	All patients, *N* = 438
Demographics	
Age (years), median (IQR)	14.19 (12.45, 15.77)
Sex, *n* (*%*)	
Female	218 (49.8)
Race/Ethnicity, *n* (*%*)^a^	
Non‐Hispanic White	31 (7.1)
Non‐Hispanic Black	103 (23.5)
Non‐Hispanic Asian	12 (2.7)
Hispanic/Latino	258 (58.9)
Other	31 (7.1)
Metabolic and liver function markers and initial treatment	
HbA1c (%), median (IQR)	8.30 (7.00, 10.70)
ALT (IU/L), median (IQR)	44.00 (24.00, 91.75)
ALT elevated, *n* (*%*)	
Yes	256 (58.4)
No	182 (41.6)
AST (IU/L), median (IQR)	36.00 (20.00, 61.25)
AST/ALT ratio, median (IQR)	0.77 (0.60, 1.00)
Alkaline phosphatase, median (IQR)	179 (116, 264)
BMI (kg/m^2^), median (IQR)	35.58 (31.27, 40.62)
BMI z‐score, median (IQR)	2.42 (2.16, 2.72)
Metformin prescribed, *n* (*%*)	
Yes	39 (8.9)
No	399 (91.1)
Fatty liver assessment (at any time)	
Referral to GI, *n* (*%*)	
Yes	63 (14.4)
Liver biopsy, *n* (*%*)	
Yes	18 (4.1)
Fatty infiltration of liver on imaging, *n* (*%*)	
Yes	88 (20.1)
No	12 (2.7)
No imaging of liver completed	338 (77.2)

*Note:* Data were reported as *n* (*%*) for categorical variables, mean (SD) for normally distributed continuous variables, and median (IQR) for non‐normally distributed continuous variables.

Abbreviations: ALT: alanine transaminase; AST: aspartate aminotransferase; BMI: body mass index; GI: gastroenterologist; HbA1c: glycated haemoglobin IQR: interquartile range; SD: standard deviation.

^a^ Three patients with missing race/ethnicity data.

Among all patients, 238 (58%) presented with elevated ALT levels at the time of diagnosis of T2D (Table 1). Of those with high ALT levels, 85 (33%) had liver imaging (ultrasound or MRI), and 80 (94%) of those with imaging had fatty infiltration seen. It is worth noting that 8 patients, despite presenting without elevated ALT levels, did have fatty infiltration of the liver on imaging (Table 2). Those patients' ALT levels ranged from 22 to 37 IU/L, with a median of 25 IU/L.

**TABLE 2:** Comparison between patients who had elevated ALT at diagnosis vs. those who did not (reference category: Patients with elevated ALT).

	Descriptive Statistics by ALT Levels at Diagnosis		Univariate Regression
	Patients with elevated ALT at diagnosis, *N* = 256	Patients with normal ALT at diagnosis, *N* = 182	Unadjusted‐OR, 95% CI
Age, median (IQR)^a^	14.25 (12.38, 15.77)	14.15 (12.58, 15.78)	1.01, (0.93, 1.10)
Sex, *n* (*%*)^b^			
Male	136 (53)	84 (46)	—
Female	120 (47)	98 (54)	0.76, (0.52, 1.11)
Race/Ethnicity, *n* (*%*)^b^			
Hispanic/Latino	188 (73)	70 (38)	—
Non‐Hispanic White	18 (7)	13 (7)	0.52, (0.24, 1.11)
Non‐Hispanic Black	29 (11)	74 (41)	**0.15, (0.09, 0.24)** ^**d**^
Non‐Hispanic Asian	8 (3)	4 (2)	0.74, (0.22, 2.55)
Other	12 (5)	19 (10)	**0.24, (0,11, 0.51)** ^**d**^
HbA1c, median (IQR)^c^	8.20 (7.10, 10.00)	9.20 (7.00, 11.68)	**0.87 (0.80, 0.94)** ^**d**^
BMI, median (IQR)^a^	35.75 (31.47, 40.62)	35.35 (30.94, 41.05)	1.00 (0.98, 1.03)
BMI z‐score, median (IQR)^a^	2.44 (2.14, 2.73)	2.42 (2.17, 2.71)	1.03 (0.67, 1.59)
Referral to GI, *n* (*%*)			
No	199 (78)	176 (97)	—
Yes	57 (22)	6 (3)	**8.40, (3.54, 19.96)** ^**d**^
Liver biopsy, *n* (*%*)			
No	238 (93)	182 (100)	—
Yes	18 (7)	—	—
Fatty infiltration on imaging, *n* (*%*)			
No	5 (2)	7 (4)	—
Yes	80 (31)	8 (4)	**14.00 (3.60, 54.46)** ^**d**^

Abbreviations: BMI, body mass index; HbA1c, glycated haemoglobin.

^a^ Independent *t*‐test.

^b^ Chi‐square test.

^c^ Wilcoxon test.

^d^ Statistically significant.

Race/ethnicity was significantly associated with ALT elevation at diagnosis. Given the Hispanic majority population, Hispanic patients were used as the reference group. Compared to Hispanic patients, non‐Hispanic Black patients had 85% lower odds of high ALT (OR 0.15, 95% CI 0.09–0.24), while those identifying as “other” race had 76% lower odds (OR 0.24, 95% CI 0.11–0.51). Non‐Hispanic White and non‐Hispanic Asians did not have significantly different odds of having a high ALT at diagnosis. Elevation of ALT at diagnosis was also associated with HbA1c at diagnosis, with each one percentage‐point increase in HbA1c corresponding to 13% decreased odds of ALT elevation (OR 0.87, 95% CI 0.80, 0.94). There was no significant difference in age and BMI at T2D diagnosis between patients with elevated ALT levels and those with normal ALT (Table 2).

**FIGURE 1:**
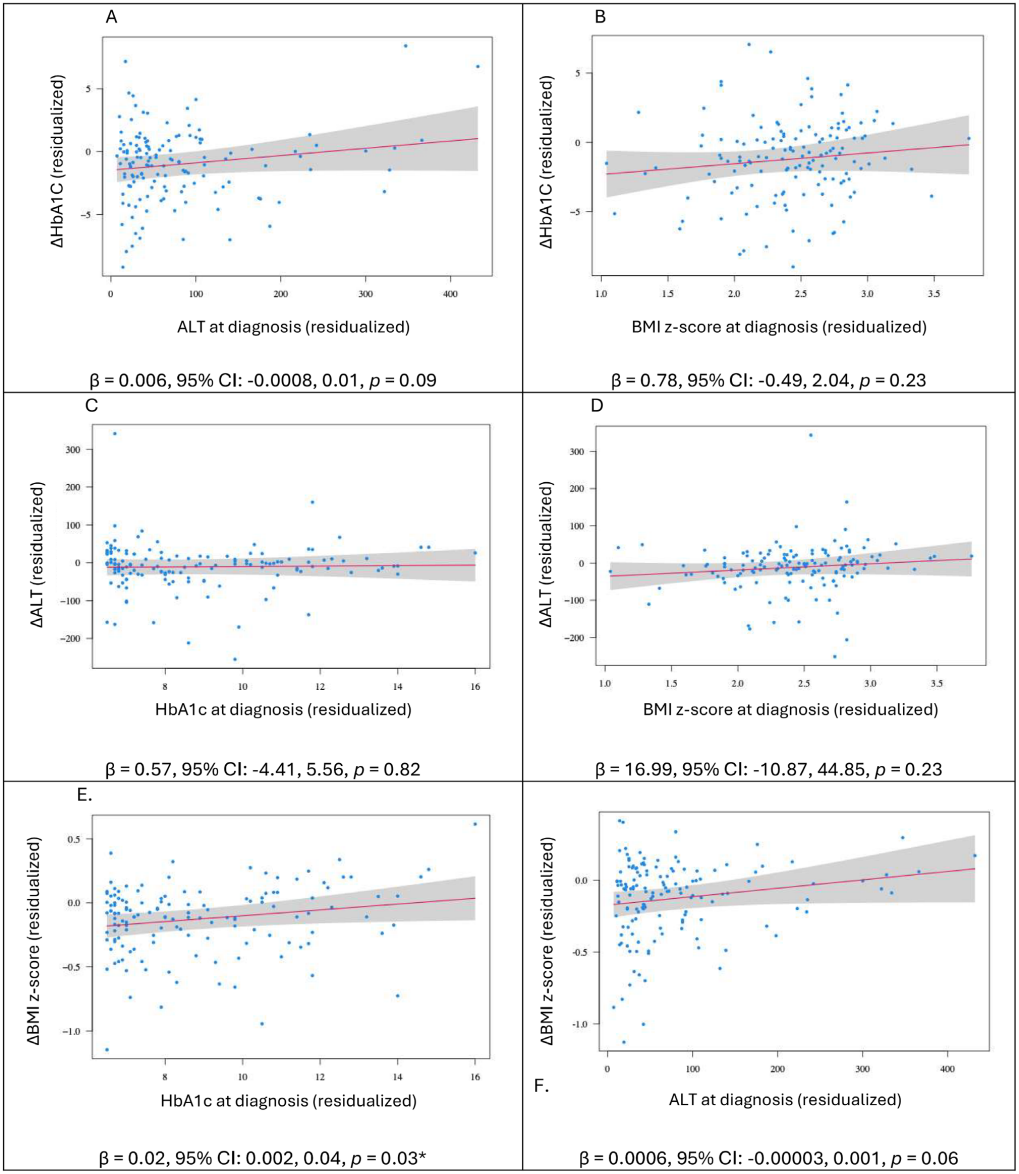
(A, B) Adjusted associations of ALT and BMI z‐score at diagnosis with the change in HbA1c over one year; (C, D) Adjusted associations of HbA1c and BMI z‐score at diagnosis with the change in ALT over one year; (E, F) Adjusted associations of HbA1c and ALT at diagnosis with the change in BMI z‐score over one year; All multiple regression models included the relevant clinical measures at diagnosis as the main predictors and were additionally adjusted for age, sex, race/ethnicity, and metformin use. *indicates statistically significant result.

Among the patients who had liver laboratory results available 9–15 months after T2D diagnosis (*n* = 141), HbA1c, ALT, AST, and BMI z‐score decreased significantly over the first year of treatment of T2D, but there was no significant change in AST/ALT ratio (Table 3). Neither ALT at diagnosis nor BMI z‐score at diagnosis was significantly associated with the one‐year change in HgbA1C (∆HbA1c) after adjusting for covariates. Similarly, BMI z‐score and HbA1c at diagnosis were not significantly associated with the one‐year change in ALT (∆ALT). In contrast, HbA1c at diagnosis was positively associated with the one‐year change in BMI (∆BMI, β = 0.02, 95% CI: 0.002–0.04), whereas ALT at diagnosis was not. The adjusted associations are illustrated in Figure 1.

**TABLE 3:** Comparison of metabolic features: T2D diagnosis and one‐year follow‐up (*N* = 141).

	Descriptive Statistics by time		Statistical Significance
	At T2D diagnosis	At one‐year follow‐ up	*p*
HbA1c (%), median (IQR)	8 (6.90, 10.50)	6.40 (5.70, 8.05)	< 0.001
ALT (IU/L), median (IQR)	46.00 (27.00, 93.00)	33.00 (20.00, 63.00)	< 0.001
ALT elevated, *n* (%)			< 0.001
Yes	92 (65)	66 (47)	
No	49 (35)	75 (53)	
AST (IU/L), median (IQR)	40.00 (24.00, 65.00)	28.00 (21.00, 41.00)	< 0.001
AST/ALT ratio, median (IQR)	0.77 (0.58, 1.00)	0.81 (0.59, 1.08)	0.27
BMI (kg/m^2^), mean (SD)	36.97 (7.28)	36.84 (7.52)	0.64
BMI z‐score, mean (SD)	2.43 (0.45)	2.33 (0.48)	< 0.001

Abbreviation: AST, aspartate transaminase.

## 4 Discussion

To our knowledge, this is the largest study exploring the prevalence of ALT elevation in youth‐onset T2D at diagnosis and the first that characterises the change in ALT elevation over the first year of T2D treatment. Overall, our study found a high prevalence of ALT elevation at diagnosis of youth‐onset T2D in a large cohort, which was similar to prior research [11]. Large differences were seen between the odds of elevated ALT at diagnosis between patients of different races and ethnicity and the confidence interval for these findings was relatively narrow. In particular, Black patients had lower odds of ALT elevation and Hispanic patients had higher odds of ALT elevation, which is consistent with prior literature [36, 37].

Surprisingly, HbA1c was negatively associated with ALT at diagnosis, though the magnitude of this association was relatively small (0.87 odds ratio). The reason behind the negative association of HbA1c and ALT at diagnosis is unclear but may be related to differences in the pathophysiology of different subgroups of T2D. While subgroups are not well characterised in youth‐onset T2D, in adult‐onset T2D, those with severe insulin resistance diabetes vs. severe insulin deficiency diabetes are known to have a higher risk for fatty infiltration of the liver [18]. Patterns of insulin resistance vs. insulin deficiency, unfortunately, are not known among our population. Over time, ALT and HbA1c generally trended down together with treatment. While ALT at diagnosis was associated with ∆BMI z‐score over time, the association was quite small and of questionable clinical significance (β = 0.0006, 95% CI: −0.00003, 0.001).

A higher ALT at diagnosis did not significantly correlate with the ∆HbA1c after one year, nor did HbA1c at diagnosis significantly correlate with ∆ALT. While the ALT did decrease over time, the prevalence of elevated ALT remained high. Clinically, earlier assessment of ALT elevation for confirmation of MASLD diagnosis for all patients with new onset T2D may be indicated, as some clinicians may currently presume these levels will decrease to normal in most patients after treatment.

The strengths of our study include a large sample size of patients with youth‐onset T2D and a real‐world estimation of the prevalence of ALT elevation as well as trends over time. Our study had several limitations. Since our study was retrospective, there can be no conclusions made about causality. There may be reporting bias for changes in HbA1c or ALT over time because of loss of patients to follow‐up. A significant number of patients were not followed from diagnosis to one year—some patients were completely lost to follow‐up, some followed at other locations, and many did not have liver testing done within the designated time period. Patients may have been more likely to have results done in this time frame for a variety of reasons: More clinical concern because of higher ALT levels at diagnosis, more significant obesity, more clinical follow‐up by family, or variation in practice among different providers. Given the wide potential reasons for attrition, it is hard to predict how this may have biased results. Furthermore, the generalisability of the findings may be limited because they were completed at a single center which may have specific referral or prescribing practices as well as patient populations that are concentrated within the area. For example, there is no significant representation of Asian or Native American populations. Due to the retrospective nature of the study, we were unable to fully assess the effect of specific treatments, such as glucagon‐like peptide agonists or metformin, that may influence liver chemistries (though metformin was reported as a categorical variable). This limitation highlights an important direction for future research to explore the potential impact of these treatments on liver chemistries and MASLD in correlation with the impact on glycemia. Given the impact of diabetes therapy on both disease states, which is not fully accounted for in our analysis, we may be seeing an attenuation of the impact of MASLD and hyperglycemia on each other. We used ALT elevation to approximate MASLD, which is not universally accepted as a biomarker for disease. Indeed, some children with MASLD will not have an increase in ALT. Additionally, the degree of ALT elevation does not always predict the severity of liver disease, so changes in ALT over time may not represent a true change in MASLD disease state [38, 39]. Therefore, our study likely underestimated the true prevalence of MASLD in this population and likely does not capture some of the associations between MASLD and other clinical variables. Additionally, pubertal status could not be adequately estimated for all patients because of inconsistent and unreliable reporting in the medical record. Finally, because of the sample size, we were unable to do significant subgroup analysis within the population to better understand the association of HbA1c and ALT within each group.

We hypothesised that ALT elevation at diagnosis would correlate with a poorer prognosis for glycemia in T2D in youth in short‐term follow‐up. While there did appear to be a small positive relationship between ALT at diagnosis and ∆HbA1c, it was not statistically significant (β = 0.006, 95% CI: −0.0008, 0.01, *p* = 0.09). The small association may be a signal of an association that would be more pronounced in a larger population, or it may indicate that work needs to be done at the subpopulation level of different subtypes of Type 2 Diabetes to see more meaningful trends. Future work in larger research cohorts can track liver disease over time to explore changes in subgroups, and more intensive translational science in small cohorts can examine changes in insulin sensitivity and resistance in liver disease progression.

## 5 Conclusions

We found that ALT elevation is common at the diagnosis of T2D in youth and generally decreases over the course of one year of treatment, but many have persistent ALT elevation. While improvement of HbA1c over time and diabetes treatment is correlated with a decrease in ALT, HbA1c level had a negative correlation with ALT at diagnosis. This study helps to characterise ALT levels in the early stages of T2D treatment and fills a gap in the literature by providing real‐world findings within this population. Future research in larger cohorts can further explore the relationship between liver disease and glycemic control over time within T2D subgroups.

## Author Contribution

**Sean DeLacey:** conceptualization (supporting); investigation (lead); data curation (supporting); methodology (lead); visualization (supporting); writing – original draft (lead); writing‐review and editing (equal). **Wenya Chen:** data curation (lead); formal analysis (lead); methodology (supporting); software (lead); visualization (lead); writing‐review and editing (equal). **Adesh Ranganna:** data curation (supporting); investigation (supporting); review and editing (equal). **Siyuan Feng:** data curation (supporting); investigation (supporting); writing – review and editing (equal). **Mark Fishbein:** conceptualization (supporting); writing – review and editing (equal). **Monica Bianco:** conceptualization (lead); investigation (supporting); methodology (supporting); supervision (lead); writing – original draft (supporting); writing – review and editing (equal).

## Funding

SD's work was partially supported by the National Institutes of Health: National Institutes of Health, Ruth L. Kirschstein National Research Service Award (T32 DK007169). MB's work was partially supported by an administrative supplement (3 R01DK118403‐02S1). SF's work was partially supported by the National Institute of General Medical Sciences: National Institutes of Health, Northwestern Medical Scientist Training Program (T32GM144295). Research involved the use of REDCap at Northwestern University Clinical and Translational Sciences (NUCATS) Institute. NUCATS is funded in part by a Clinical and Translational Science Award (CTSA) grant from the National Institutes of Health (NIH) UL1TR00142. Research was also supported by a voucher from the Stanley Manne Children's Research Institute at Ann & Robert H. Lurie Children's Hospital of Chicago.

## Conflicts of Interest

The authors declare no conflicts of interest.

## Supporting information


**Table S1:** Definitions of Variables.

## Data Availability

The data that support the findings of this study are available from the corresponding author upon reasonable request.
