# Supplementary material for: Alanine Aminotransferase Elevation at Diagnosis of Youth‐Onset Type 2 Diabetes: Prevalence, Predictors, and One‐Year Outcomes
Source: Endocrinol Diabetes Metab. 2026 Apr 11;9(3):e70211. doi: 10.1002/edm2.70211 (PMC13070023; doi:10.1002/edm2.70211)
Supplement: Supplementary file 1 — Table S1: Definitions of Variables. [file EDM2-9-e70211-s001.docx]

| Supplementary Table 1: Definitions of Variables | |
| --- | --- |
| Variable | Definition |
| Type 2 Diabetes | Glycated hemoglobin (HbA1c) greater than or equal to 6.5% (48 mmol/mol), OR  oral glucose tolerance test results (fasting >126 mg/dL OR 2 h > than 200 mg/dL), OR  a random blood glucose of >200 mg/dL with consistent symptomatology |
| ALT elevation | ALT> 1.5 times the upper limit of normal based on sex (22.1 IU/L for females and 25.8 IU/L for males) |
